# Rearing bucks isolated from females affects negatively their sexual behavior when adults

**DOI:** 10.21451/1984-3143-AR2017-0047

**Published:** 2018-08-16

**Authors:** Lorena Lacuesta, Julia Giriboni, Agustín Orihuela, Rodolfo Ungerfeld

**Affiliations:** 1 Departamento de Fisiología, Facultad de Veterinaria, Universidad de la República, Montevideo11600, Uruguay.; 2 Facultad de Ciencias Agropecuarias, Universidad Autónoma de Morelos, Cuernavaca 62209, México.

**Keywords:** goat, socio-sexual signals, sexual performance

## Abstract

In some domestic ruminants, contact with females is necessary for normal development of sexual behavior. The aims of this experiment were to determine if rearing bucks isolated from does affects negatively their sexual behavior when adults, and if this negative effect is overcome after four short contacts with females. Sixteen Saanen male kids were maintained during one year in two groups: kids reared in permanent direct contact with four adult goats (FEM; n = 7), and kids that remained isolated from females (ISO; n = 9). When bucks were 12 mo-old, females were removed and both groups were joined in the FEM pen. Nine months later all bucks were individually exposed four times to estrual females for 20 min every 10 days, recording courtship and mounting behaviors. Bucks that were reared with females displayed more courtship behaviors, ejaculations and total mounts (mount attempts, mounts with and without ejaculation; P < 0.0001 for all) than ISO bucks. The combined effect of number of bucks that ejaculated and the time at which they first ejaculated in the first and second tests favored FEM bucks (P < 0.03). It was concluded that the lack of contact with females during the rearing period affects negatively adult bucks’ sexual performance, an effect that could not be overcome after repeated exposures to estural does.

## Introduction

In sheep, heterosexual contact is necessary for normal male sexual development and behavior (Zenchak *et al.*, 1980; Casteilla *et al*., 1987 ). In this sense, male lambs reared in permanent contact with females have a greater testicular volume and display a more intense sexual behavior than lambs reared isolated from them ( Illius *et al*., 1976 ; Katz *et al*., 1988 ). Katz *et al*. (1988) showed that rams reared with adult females during their pre-pubertal period mounted females more times than rams reared in all-male groups. In the same direction, rams that display high levels of courtship and mounting toward other males during rearing have low sexual interest in females when adults (Zenchak and Anderson, 1981). Therefore, the socio-sexual environment in which males are reared has a strong influence on the sexual behavior displayed when adults.

However, the importance of social environment during development differs according to the species. In this sense, contact with females during their pre-pubertal period has no effect on bulls ( Lane *et al*., 1983 ; Price and Wallach, 1990 ; Borg *et al*., 1993 ) and boars’ sexual performance and libido ( Hemsworth *et al*., 1977 ). In bucks, while a brief and acute exposure to estural goats when males were yearlings did not modify their sexual performance when adults ( Price *et al*., 1998 ), permanent contact with adult does during their pre-pubertal development has short lasting positive effects in reproductive traits ( Lacuesta *et al*., 2015 ). It is also interesting that bucks that were reared isolated from females recognize other males as sexual partners and thus, are more predisposed to display homosexual behaviors than bucks that were reared with females ( Ungerfeld *et al*., 2013 ). Thus, the lack of contact with females during their pre-pubertal period affects the sexual display of bucks when adults.

Therefore, the aims of this experiment were to determine if sexual behavior of bucks that were reared isolated from does is negatively affected when adults, and if so, if this negative effect is overcome after four contacts with females.

## Materials and methods

### Animals and management

The experiment was performed in the Facultad de Veterinaria, Universidad de la República (Uruguay, 35°S) during the breeding season (April) with the same 16 male Saanen bucks previously used in Lacuesta *et al*. (2015) . Briefly, all kids were fathered by the same buck, weaned 24 h after birth, and artificially reared with milk supplement in a heterosexual group until 20 days of age. At that age, male kids were placed in one of two groups homogeneous in body weight, each allocated in a 17 X 10 m pen. While FEM male kids (n = 7) were reared in permanent direct contact with four adult does, ISO male kids (n = 9) remained isolated from females (minimum distance 5000 m) until they were 12 mo-old. At that age does were removed from the FEM group, and one week later, both groups were joined, remaining together until sexual tests were performed when bucks were 21 mo-old. After does were taken out from the FEM group, the pregnant diagnosis was done resulting positive in all does, so FEM males had sexual experience during their pre-pubertal development. All animal management was approved by the Comisión Honoraria de Experimentación Animal (CHEA-Ethical Committee for Experiments with Animals) of the Facultad de Veterinaria.

The sexual tests were performed when bucks were 21 mo-old. At that time, FEM bucks weighed 48.4 ± 1.8 kg and ISO bucks 49.8 ± 1.2 kg (mean ± SEM). During the experiment, the males received lucerne hay and concentrate according to the nutritional requirement for growth, and water ad libitum.

### Sexual behavior tests

Bucks were individually exposed in random order to an estural unknown doe free in the pen on 4 occasions separated by 10 days each. The tests were performed in a different pen (5 X 4 m) from where bucks were allocated, located 300 m away. The female was induced into estrus by a hormonal treatment (5 days of intravaginal sponge impregnated with medroxiprogesterone acetate and injections of 1.5 mg of estradiol benzoate at the time of withdrawal). Once the sexual test ended, each buck was transported back to another pen (3000 m away from where bucks were held) so bucks that had finished the test did not have any contact with bucks that were still not tested. The sexual behavior was always recorded by the same observer, who remained outside the pen, without interfering with bucks’ behavior. The number of courtship (anogenital sniffs, flehmen and lateral approaches) ( Shackleton and Shank, 1984 ) and mounting (mount attempts, mounts without and with ejaculation) behaviors were recorded during 20 min in each test, and the number of total mounts/ejaculations and ejaculations/total mounts were calculated. In addition, time to the first ejaculation in each test (latency) was recorded.

### Statistical analysis

The frequency of each behavior displayed by FEM or ISO bucks in each test was compared using a Glimmix procedure assuming a Poisson distribution. The main effects considered in the model were the treatment, the number of test and the interaction between the treatment and the number of test. The latency to first ejaculation and the number of males that ejaculated from each group were compared combined with a survival test. Data are presented as mean ± SEM.

## Results

### Sexual behavior

**Figure 1:**
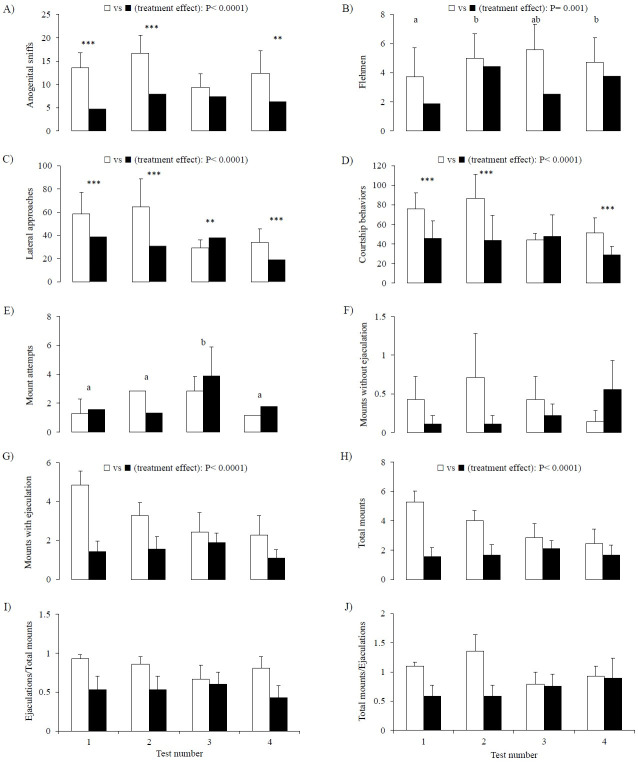
Number of (A) anogenital sniffs; (B) flehmen; (C) lateral approaches; (D) courtship behavior (sum of all courtship behaviors); (E) mount attempts; (F) mounts without ejaculation; (G) mounts with ejaculation; (H) total mounts; (I) ejaculations/total mounts relationship and (J) total mounts/ejaculations relationship in four tests with an estrous doe performed by adult bucks that were reared with (white columns) or without continuous contact with females (black columns) until they were 1 year old. Different letters indicate significant differences between time points for each group. Differences between groups for each time point are shown with asterisks: ** P < 0.005; *** P < 0.0001. Treatment effects are shown as □ FEM vs. ■ ISO P < 0.0001.

The number of anogenital sniffs, flehmen and lateral approaches was greater in FEM than ISO bucks (P < 0.0001; P = 0.001; P < 0.0001, respectively) and varied with the number of test (P < 0.009; P = 0.03; P < 0.0001, respectively) ( Fig. 1A , 1B and 1C respectively). There was an interaction between treatment and number of test in anogenital sniffs and lateral approaches (P = 0.02 and P < 0.0001, respectively). Bucks that were reared with females displayed more anogenital sniffs in the first, second and fourth tests (test 1 and 2: P < 0.0001; test 4: P < 0.0002), and more lateral approaches in all the tests (test 1, 2 and 4: P < 0.0001; test 3: P < 0.005) than ISO bucks. The number of courtship behaviors (sum of anogenital sniffs, flehmen and lateral approaches; Fig. 1D ) was also greater in FEM than ISO bucks (P < 0.0001), varied with number of test (P < 0.0001) and there was an interaction between treatment and number of test (P < 0.0001). It was greater in FEM than in ISO bucks in the first, second and fourth tests (P < 0.0001 for all tests).

The number of mount attempts ( Fig. 1E ) was not affected by the treatments, but varied with time (P = 0.001): it was greater in the third than in all the other tests, and there was a tendency for an interaction between treatment and number of test (P = 0.09). The number of mounts without ejaculation was not affected by treatment or number of test ( Fig. 1F ). However, the number of mounts with ejaculation and the total number of mounts were greater in FEM than in ISO bucks (P < 0.0001, for both; Fig. 1G and 1H , respectively). There were no effects of treatment, number of test or interaction between treatment and number of test on ejaculations/total mounts and total mounts/ejaculations ( Fig. 1I and 1J , respectively).

The combined effect of the number of bucks that ejaculated and it’s latency (survival test), favored FEM bucks in the first and second tests (P < 0.03; Table 1 ).

**Table 1:** Number of adult bucks that ejaculated and interval to the first ejaculation in four tests with an estrous doe during a 20 min pen test, performed by adult bucks reared with (FEM) or without female contact (ISO) during their pre-pubertal period. The P value corresponds to the Survival test.

Test	1		2		3		4	
Group	Number of bucks	Time (s)	Number of bucks	Time (s)	Number of bucks	Time (s)	Number of bucks	Time (s)
FEM	7/7	46.0 ± 15.3	7/7	43.8 ± 11.5	6/7	39.0 ± 12.6	6/7	84.3 ± 45.8
ISO	5/9	159.0 ± 109.6	5/9	196.0 ± 159.0	6/9	69.2 ± 39.1	5/9	61.6 ± 33.2
P	0.02		0.03		ns		ns	

## Discussion

Adult bucks that were reared isolated from females during their pre-pubertal period displayed poorer sexual behavior toward estural does than bucks reared in close contact with them. This included a reduction in the courtship and mounting behaviors and a greater latency from less males that ejaculated in the first two tests. These differences were evident even although both groups of males remained isolated from females during 9 mo before the study began, reinforcing and expanding previous concepts on the great importance that the socio-sexual environment in which an animal is reared has on its adult sexual behavior. In the same direction, Ungerfeld *et al*. (2013) also reported that bucks that were reared isolated from females display greater sexual behavior toward other males than those reared with females, suggesting that the former have a reduced ability to discriminate possible sexual partners according to their gender. Overall, the social environment in which male goats were reared had great consequences on their sexual behavior as adults.

Male sexual behavior has direct consequences on reproductive success, and as a consequence, in the flock fertility ( Tilbrook and Cameron, 1990 ). Although this study only tested the difference between bucks reared in contact or isolated from females, kids are commonly reared with different degrees of contact with females (different male:female proportions, density of animals, etc). Therefore, it is interesting to speculate that differences in intensity of contact with females during bucks’ rearing may partially explain individual differences in sexual behavior (see review: Katz, 2008 ), field breeding efficiency, and thus, in their offspring.

The poorer sexual behavior displayed by bucks that were reared in all male groups is in agreement with similar results previously reported in yearling rams ( Casteilla *et al*., 1987 ; Katz *et al*., 1988 ). In bucks, Price *et al*. (1998) reported that short exposures of post-pubertal male goats to estural females during their first year of life did not enhance their sexual performance when they achieve their adultness. Considering that Imwalle and Katz (2004) reported that sexually-naïve bucks require only one serving capacity test before they attain full sexual performance, in this experiment it was demonstrated that the lack of contact with females during males’ growth has sustained effects when adults, that cannot be overcome during at least four contacts. In addition, sexual behavior did not show a specific evolution pattern over time. Only the frequencies of mount attempts and flehmen varied with time independently from the treatments, but did not follow a clear pattern. On the other hand, continuous contact with estrual goats stimulates the reproductive activity of adult bucks ( Giriboni et al., 2017 ). Although this study was not continued for more time, probably the lower intensity of the display of sexual behavior of the bucks that were reared isolated from females would be sustained over time.

Overall, it was concluded that the lack of contact with females during the rearing period affects negatively adult bucks’ sexual performance, effect that could not be overcomed after repeated exposures to estural does.
